# Clinical Significance of Septal Malalignment for Transcatheter Closure of Atrial Septal Defect

**DOI:** 10.1155/2020/6090612

**Published:** 2020-03-03

**Authors:** Yoichi Takaya, Teiji Akagi, Koji Nakagawa, Rie Nakayama, Takashi Miki, Nobuhisa Watanabe, Norihisa Toh, Hiroshi Ito

**Affiliations:** ^1^Department of Cardiovascular Medicine, Okayama University Graduate School of Medicine Dentistry and Pharmaceutical Sciences, Okayama, Japan; ^2^Division of Medical Support, Okayama University Hospital, Okayama, Japan

## Abstract

**Background:**

Septal malalignment is related to erosion and device embolization in transcatheter closure of atrial septal defect (ASD), but limited information is available.

**Objectives:**

This study aimed to assess clinical significance of septal malalignment and to determine appropriate evaluation of ASD diameter, including the selection of device size.

**Methods:**

Four hundred and seventeen patients with ASD who underwent transcatheter closure were enrolled. Septal malalignment was defined as separation between the septum primum and the septum secundum on transesophageal echocardiography.

**Results:**

One hundred and eighty-four patients had septal malalignment. The frequency of septal malalignment increased with age reaching around 50% in adult patients. Septal malalignment was related to aortic rim deficiency. The distance of separation between the septum primum and the septum secundum was 5 ± 2 mm (range, 1–11 mm). In patients with septal malalignment, the ASD diameter measured at the septum primum was 19 ± 6 mm, while the ASD diameter measured at the septum secundum was 16 ± 6 mm. There was a difference of 4 ± 2 mm (range, 0–8 mm) between the ASD diameter measured at the septum primum and that measured at the septum secundum. For transcatheter closure, the Amplatzer Septal Occluder device size 2-3 mm larger and the Occlutech Figulla Flex II device size 4–7 mm larger than the ASD diameter measured at the septum primum were frequently used. During the study period, erosion or device embolization did not occur in all of the patients.

**Conclusions:**

Septal malalignment is highly prevalent in adult patients with aortic rim deficiency. The measurement of ASD diameter at the septum primum can be valuable for the selection of device size in patients with septal malalignment.

## 1. Introduction

Transcatheter closure has been established as an effective treatment for atrial septal defect (ASD) and has become an alternative to surgical closure [1–6]. Although transcatheter closure is safe, serious complications such as erosion and device embolization occur. The etiology of these complications is multifactorial. A deficient aortic rim and over- and/or undersized device use have been implicated as risk factors [7]. Recently, some studies have focused on the relationship of septal malalignment with erosion and device embolization [8–10]. In septal malalignment, the septum primum is malaligned toward the left atrial side and is separated from the septum secundum. To prevent the complications, the recognition of the presence of septal malalignment is important. Accurate evaluation of ASD diameter is also necessary. To stably deploy the device, the left atrial disc is needed to be placed at the septum primum, and the right atrial disc is needed to be placed at the septum secundum. Thus, ASD diameter is considered to be measured at the point of the septum primum. However, septal malalignment has not been fully investigated. Therefore, this study aimed to assess clinical significance of septal malalignment and to determine appropriate evaluation of ASD diameter, including the selection of device size.

## 2. Methods

### 2.1. Study Population

The study population comprised 417 consecutive patients with ASD who underwent transcatheter closure from November 2011 to September 2018 in our institution. Indications for transcatheter closure were a significant left-to-right shunt, right ventricular volume overload, and/or clinical symptoms of heart failure. Patients with multifenestrated defects, other congenital heart disease, or the status of postsurgical repair for defect were excluded. All patients gave written informed consent for the procedure. The study was approved by the ethics committee of our institution.

### 2.2. Septal Malalignment

**Figure 1:**
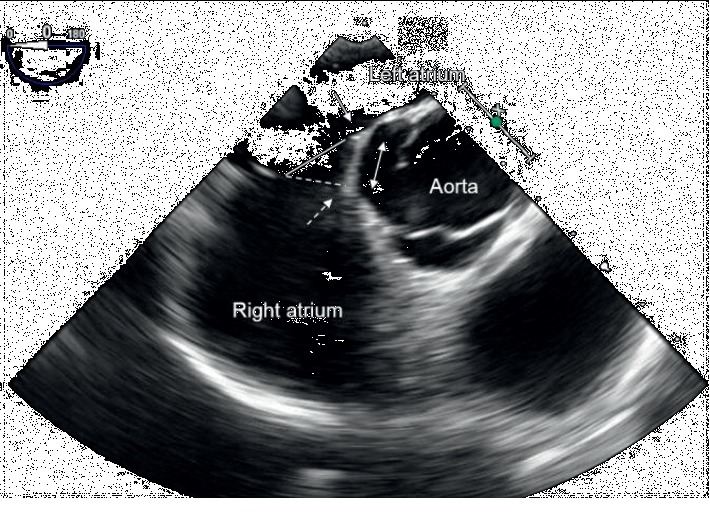
Transesophageal echocardiography showing septal malalignment. The septum primum attached to the aorta is malaligned toward the left atrial side (solid arrow). The septum primum is separated from the septum secundum (dotted arrow). The defect surface of the septum primum (solid line) is different from that of the septum secundum (dotted line). The distance of separation between the septum primum and the septum secundum is measured (double arrow).

All patients underwent transesophageal echocardiography (TEE) (iE33; Philips Medical Systems, Andover, MA, USA) before and during the procedure. TEE evaluated ASD morphology with sweeping from 0 to 180 degrees, including imaging at 0, 30, 45, 60, 75, 90, 120, 135, and 150 degrees. Septal malalignment was defined as the separation between the septum primum and the septal secundum on the aortic wall. Septal malalignment was evaluated in the short-axis aortic view from 0 to 45 degrees. To detect the presence of septal malalignment, we investigated whether the septum attached to the aorta was separated on TEE. When the septum primum was malaligned toward the left atrial side and the separation of the septum primum and the septum secundum was observed [10], the patient was diagnosed as having septal malalignment. The ASD diameter was determined at the point of the septum primum but not at the point of the septum secundum (Figure 1). For the severity of septal malalignment, the distance of separation between the septum primum and the septum secundum was assessed.

### 2.3. Transcatheter Closure

Transcatheter closure was performed under TEE guidance as described previously [11] using the Amplatzer Septal Occluder device (Abbott, Chicago, IL, USA) and the Occlutech Figulla Flex II device (Occlutech GmbH, Jena, Germany). The device size was selected on the basis of the maximal ASD diameter evaluated by TEE.

### 2.4. Study Design

This was an observational cohort study. First, we investigated the incidence and severity of septal malalignment and the morphological characteristics related to septal malalignment. Second, we assessed whether the evaluation of ASD diameter at the point of the septum primum was appropriate, including the selection of device size. Because septal malalignment was observed from 0 to 45 degrees on TEE, 329 patients who had the maximal diameter of ASD within these degrees were included in this analysis.

### 2.5. Statistical Analysis

Data are presented as mean ± standard deviation for continuous variables and as number and percentage for categorical variables. Differences between the two groups were analyzed by the *t*-test and Mann–Whitney *U* test for continuous variables and the *χ* [2] test for categorical variables. Relationship between the severity of septal malalignment and hemodynamics was assessed by Pearson's correlation coefficient. Statistical analysis was performed with JMP version 14.2 (SAS Institute Inc., Cary, NC, USA), and significance was defined as a value of *P* < 0.05.

## 3. Results

### 3.1. Clinical Characteristics

**Figure 2:**
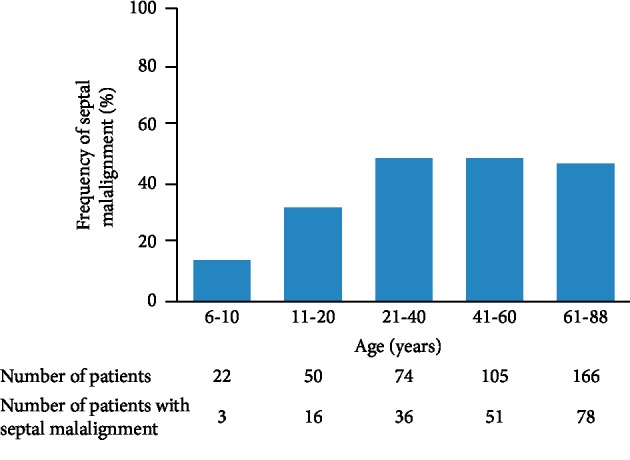
Frequency of septal malalignment. The frequency of septal malalignment according to age is shown.

**Figure 3:**
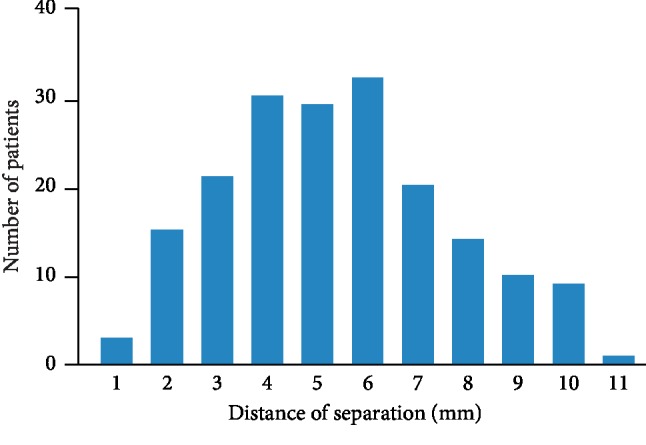
Severity of septal malalignment. The distance of separation between the septum primum and the septum secundum in patients with septal malalignment is shown.

The mean age of the patients was 49 ± 22 years (range, 6–88 years). Septal malalignment was observed in 184 (44%) patients. Comparisons of clinical characteristics between patients with septal malalignment and those without septal malalignment are shown in Table 1. Patients with septal malalignment were older than those without septal malalignment. Aortic rim deficiency, which was defined as <5 mm, was observed more frequently in patients with septal malalignment than in those without septal malalignment. Pulmonary-to-systemic blood flow ratio was higher in patients with septal malalignment than in those without septal malalignment. The frequency of septal malalignment according to age is shown in Figure 2. Almost 50% of patients ≥21 years old had septal malalignment, while septal malalignment was observed in 16 (32%) of 50 patients aged 11–20 years and in three (14%) of 22 patients aged ≤10 years. This frequency increased with age until adulthood. The distance of separation between the septum primum and the septum secundum is shown in Figure 3. The mean distance of separation was 5 ± 2 mm (range, 1–11 mm). The distance of separation of ≥10 mm was observed in some patients. The distance of separation was related to pulmonary-to-systemic blood flow ratio (*R* = 0.14, *P* < 0.001).

### 3.2. Evaluation of Atrial Septal Defect Diameter

**Figure 4:**
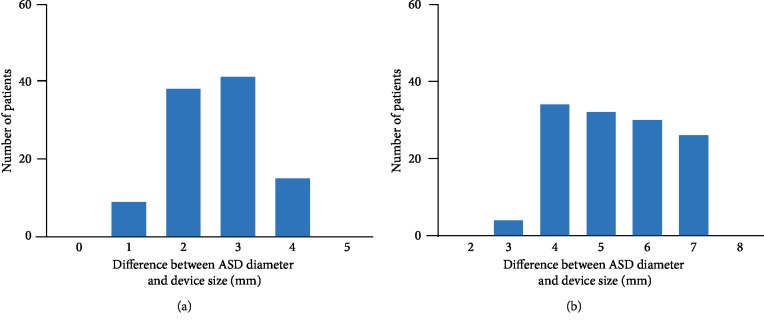
Difference between the ASD diameter and the device size in patients with septal malalignment. The difference between the ASD diameter measured at the septum primum and Amplatzer Septal Occluder device size (a) and the Occlutech Figulla Flex II device size (b). ASD, atrial septal defect.

Among 329 patients who had the maximal diameter of ASD from 0 to 45 degrees, 166 patients had septal malalignment. The ASD diameter and the device selection in patients with septal malalignment are shown in Table 2. The ASD diameter measured at the septum primum was 19 ± 6 mm, while the ASD diameter measured at the septum secundum was 16 ± 6 mm. There was a difference of 4 ± 2 mm (range, 0–8 mm) between the ASD diameter measured at the septum primum and that measured at the septum secundum. The Amplatzer Septal Occluder device was used in 103 patients with septal malalignment. The device size was 22 ± 7 mm. The difference between the device size and the ASD diameter measured at the septum primum was 2 ± 1 mm, and the device size 2-3 mm larger than the ASD diameter was frequently used (Figure 4(a)). The Occlutech Figulla Flex II device was used in 63 patients with septal malalignment. The device size was 24 ± 5 mm. The difference between the device size and the ASD diameter measured at the septum primum was 5 ± 1 mm, and the device size 4–7 mm larger than the ASD diameter was frequently used (Figure 4(b)). During the study period, erosion or device embolization did not occur in all of the patients.

## 4. Discussion

The major findings of the present study were (1) septal malalignment was frequently observed, (2) the incidence of septal malalignment increased with age until adulthood, (3) septal malalignment was related to aortic rim deficiency, and (4) the measurement of ASD diameter at the septum primum on TEE and the selection of device size based on the ASD diameter were appropriate in patients with septal malalignment. To the best of our knowledge, this is the first study to show the clinical significance of septal malalignment, including the appropriate evaluation of ASD diameter for transcatheter closure.

### 4.1. Clinical Importance of Septal Malalignment

Serious complications such as erosion and device embolization are rare but fatal. With regard to ASD morphology, a deficient rim increases the risk of these complications [7, 12] but is a common finding [13]. Therefore, other morphological factors in addition to a deficient rim may be important. Recently, septal malalignment has been proposed as a novel risk factor [8, 9]. Although the aortic rim is usually in the middle of the aorta in the short-axis view on TEE, the septum primum attached to the aorta is malaligned toward the left atrial side in septal malalignment. The septum primum and the septum secundum are separated, resulting in a difference in defect surfaces of the septum primum and the septum secundum. At the time of transcatheter closure, septal malalignment could cause a change in the device axis angle against the aorta, inducing the potential for pushing the device disc to the aorta and instability of deploying the device. This leads to the occurrence of erosion and device embolization. We previously reported one patient who had erosion 3 days after the procedure [10]. The patient had septal malalignment combined with aortic rim deficiency. Additionally, we experienced two patients for whom the device was not embolized but was unstably deployed. In both patients, we did not notice that the septum primum was malaligned toward the left atrial side on TEE during the procedure. This led to underestimation of the maximal ASD diameter, resulting in undersized device use. Therefore, the recognition of septal malalignment on TEE was considered to be important in performing transcatheter closure. However, limited information is available regarding septal malalignment. Septal morphology has not been well evaluated by TEE, even in the studies that assessed the risk factors of erosion and device embolization [13–15].

The present study showed that septal malalignment was not uncommon in adult patients with ASD. Septal malalignment was related to aortic rim deficiency. The severity of septal malalignment was widely varied. By focusing on the septum primum and the septum secundum attached to the aorta during TEE, 1 or 2 mm septal malalignment could be assessed. Our results are useful when evaluating ASD morphology for deploying the device stably in transcatheter closure. Additionally, the present study found that the frequency of septal malalignment increased with age until adulthood. The severity of septal malalignment was related to pulmonary-to-systemic blood flow ratio. Left atrial size is determined by body size. Left atrium enlarges in response to volume overload [16]. Based on these findings, septal malalignment may be a secondary change associated with the dilatation of the left atrium which is induced by chronic volume overload due to ASD.

### 4.2. Evaluation of Septal Malalignment for Transcatheter Closure

The accurate measurement of ASD diameter on TEE, leading to the optimal selection of device size, is essential to perform transcatheter closure. However, no studies have focused on how to evaluate the ASD diameter in patients with septal malalignment. To stably deploy the device, the device must enclose the septum primum and the septum secundum in between both discs, without either the disc being wedged in between the two septa. If the device encloses only the septum primum or the septum secundum, either the disc wedges into the aortic wall, leading to an increased risk for erosion and device embolization. In the present study, the ASD diameter was measured at the point of the septum primum on TEE in patients with septal malalignment, and the device size was selected based on the ASD diameter. Although our study population was relatively high risk, erosion or device embolization did not occur, indicating that the evaluation of ASD diameter was appropriate. Furthermore, the present study revealed that the device size 2-3 mm larger than the ASD diameter in the Amplatzer Septal Occluder device and 4–7 mm larger than the ASD diameter in the Occlutech Figulla Flex II device can work well in the range of defects we occluded.

### 4.3. Clinical Implications

Transcatheter closure has become an alternative to surgical closure because of less invasiveness and a shorter hospital stay [3–5]. Transcatheter closure should be performed without any complications. To achieve this goal, accurate evaluation of ASD morphology by TEE and the optimal selection of device size are essential. The present study indicates that the recognition of the presence of septal malalignment is important, especially in adult patients with aortic rim deficiency. In patients with septal malalignment, the ASD diameter should be measured at the septum primum but not at the septum secundum, which results in the optimal selection of device size. The present study provides evidence for the therapeutic strategy in patients with ASD combined with septal malalignment.

### 4.4. Study Limitations

There are limitations in the present study. First, this study was a small number of patients to assess the association between septal malalignment and the occurrence of erosion and device embolization. Large studies are required to confirm our findings. Second, the follow-up period was not long. Finally, there was selection bias because patients who underwent transcatheter closure were enrolled. Severe septal malalignment which could not be performed transcatheter closure might not be included.

## 5. Conclusions

Septal malalignment is highly prevalent, especially in adult patients with aortic rim deficiency. The measurement of ASD diameter at the septum primum on TEE can be valuable for the selection of device size in patients with septal malalignment.

## Figures and Tables

**Table 1:** Clinical characteristics.

	Malalignment (+) (*n* = 184)	Malalignment (−) (*n* = 233)	*P* value
Age (years)	52 ± 19	46 ± 24	0.02
Female	104 (57%)	144 (62%)	0.28
Aortic rim deficiency	173 (94%)	135 (58%)	<0.001
Pulmonary-to-systemic blood flow ratio	2.4 ± 0.9	2.1 ± 0.7	<0.001
Pulmonary arterial pressure (mmHg)	18 ± 7	18 ± 7	0.94

Data are presented as mean ± standard deviation.

**Table 2:** ASD diameter and device size in 166 patients with septal malalignment.

ASD diameter at the septum primum (mm)	19 ± 6
ASD diameter at the septum secundum (mm)	16 ± 6
Amplatzer Septal Occluder device	103 (62%)
Device size (mm)	22 ± 7
Occlutech Figulla Flex II device	63 (38%)
Device size (mm)	24 ± 5

Data are presented as mean ± standard deviation. ASD, atrial septal defect.

## Data Availability

The data used to support the findings of this study are included within the article.

## Supplementary Materials

Supplementary Materials Transesophageal echocardiography showing septal malalignment.
